# Exploring the breed-specific variations in meat quality attributes and gene expression patterns associated with tenderness, intramuscular fat, and fatty acid composition profiles in nellore and deccani sheep breeds of India

**DOI:** 10.5713/ab.250854

**Published:** 2026-04-02

**Authors:** Navya Pothireddy, M. R. Vishnuraj, K. Vijaya Rachel, P. Baswa Reddy, G. Ajay, K. Shiva Shankar, I. Krishnachaithanya, N. Aravind Kumar, S. B. Barbuddhe

**Affiliations:** 1 Department of Life Science (Biochemistry), GITAM University, Visakhapatnam, India; 2 ICAR-National Meat Research Institute, Hyderabad, India

**Keywords:** Fatty Acid Composition, Intramuscular Fat Content, Meat Quality, Meat Quality Regulating Genes, Nellore and Deccani

## Abstract

**Objective:**

This study aimed to investigate the meat quality attributes and expression patterns of meat quality-regulating genes of two Indian indigenous sheep breeds.

**Methods:**

Meat quality indicators and expression patterns of four candidate genes (*CAST*, *CAPN*, *CEBPB*, and *SCD*), involved in meat tenderness, intramuscular fat (IMF), and fatty acid (FA) composition in the *longissimus dorsi* muscle of healthy rams of Nellore (n = 4) and Deccani (n = 4) sheep breeds aged between 9 and 12 months, were measured.

**Results:**

The Nellore breed exhibited lower cooking loss, pH, moisture, and shear force, and higher protein. The Nellore breed exhibited higher crude protein content (p<0.05), while the Deccani breed showed higher crude fat content (p<0.05) and moisture (p<0.05). Further, FA composition varied among breeds, with the Deccani breed exhibiting a healthier profile than the Nellore breed. Relative expression of the *CAST*, *SCD*, and *CEBPB* genes was 5.51, 3.04, and 3.08-fold higher in the Deccani breed (p<0.05) compared to the Nellore breed, respectively. Significant correlations were observed between gene expression and meat quality traits. The IMF and FA composition demonstrated a strong positive correlation (p<0.05) with CEBPB and SCD expression. Moreover, in the Nellore breed, Warner-Bratzler shear force exhibited a strong negative correlation (p<0.05) with CAPN expression.

**Conclusion:**

This study demonstrated clear differences between the two sheep breeds evaluated under extensive feeding systems. The Deccani breed offers superior IMF and flavour potential, in addition to a high concentration of beneficial FAs. On the other hand, the Nellore breed’s balanced proteolytic activity and leaner composition made it favourable for tenderness.

## INTRODUCTION

India is largely an agrarian nation, and livestock forms the backbone of rural livelihoods. Among them, indigenous sheep hold special importance because of their multiple uses, such as providing meat, wool, skins, manure, and, in some cases, even milk [1]. According to the 20^th^ livestock census [2] 74.26 million sheep were reported, a 14.1% increase from the 2012 census, underlining their growing significance in livestock farming by contributing about 13.8% of India’s livestock population. Nellore is ranked 1st in terms of population, with a 20% share, followed by Bellary, Marwari, Deccani, Kenguri, and Mecheri among indigenous registered breeds [1]. The extensive geographic distribution and rich genetic resources of Indian indigenous sheep breeds, along with various adaptive pressures, have fostered the genetic and phenotypic diversity in Indian local sheep. These variations lead to notable differences in growth rates and meat quality among Indian sheep populations [3].

As mutton consumption continues to grow in India, consumer preference is shifting toward superior-quality mutton, resulting in an insufficient supply. Despite slower growth rates, they are recognised for their favourable meat quality traits. To objectively evaluate mutton quality and palatability, it is essential to conduct comparative studies on the nutritional and sensory qualities of meat. The quality of meat is shaped by a wide range of complex interactions between genetic and environmental factors, including breed, age, gender, and feeding management. Meat quality is a pivotal factor influencing health and consumer preference, encompassing both nutritional and sensory attributes. From a nutritional standpoint, meat is a valuable source of bioavailable protein, minerals, vitamins, and essential amino acids [4]. Crucially, its fatty acid composition largely determines its health implications and also contributes to sensory characteristics. Previous studies have indicated that FA profiles are significantly influenced by breed, with specific breeds exhibiting unique compositions that impact overall nutritional value and sensory attributes [5–7]. Beyond these nutritional aspects, textural quality is equally important. Studies consistently demonstrate that the Warner-Bratzler shear force (WBSF), a key indicator of meat tenderness, is significantly affected by the breed [5,8–10]. Furthermore, the proximate composition also demonstrated notable variation among different sheep breeds [8,11].

However, while phenotypic assessments provide valuable insights, meat quality is also regulated by molecular pathways controlling muscle proteolysis, lipid metabolism, and adipogenesis. Candidate genes such as calpastatin (CAST) [12–14] and calpain (CAPN1) are central to the proteolytic system that governs postmortem muscle degradation and tenderness [15]. The stearoyl-CoA desaturase (SCD) gene influences FA composition by converting saturated fatty acids (SFAs) into monounsaturated fatty acids (MUFAs), thereby improving the nutritional quality of meat lipids [16]. Similarly, CCAAT/enhancer-binding protein beta (CEBPB) plays a key role in adipocyte differentiation and intramuscular fat (IMF) deposition, which enhances juiciness and flavour [17]. These genes have been extensively studied in sheep, goat and cattle for their association with tenderness, IMF content, and fatty acid profile [18–20].

Understanding the genetic regulation of meat quality traits and carcass characteristics of indigenous Indian sheep breeds is of great significance, in light of India’s socioeconomic transformation and evolving consumer demands. However, comparative studies of carcass traits and mutton quality among typical breeds of diverse sheep across India have been limited thus far. To our knowledge, no data have yet been reported in the scientific literature pertaining to the meat quality traits and associated gene expression markers in these two prominent Indian sheep breeds. Based on these gaps, the present work was designed to explore meat quality traits and carcass characteristics in Nellore and Deccani sheep, and to evaluate the expression of CAST, CAPN1, SCD, and CEBPB, thereby elucidating their role in regulating breed-specific differences in tenderness, fat deposition, and fatty acid composition. The objective of this study is to present the mutton quality situation of selected Indian indigenous sheep breeds and provide a reference for the breeding plan of specialised mutton production breeds.

## MATERIALS AND METHODS

### Animal management and slaughter

Eight healthy rams from the Deccan plateau region in India were selected and divided into two groups: one comprised four Deccani sheep, and the other four sheep from the Nellore breed. The animals, aged between 9–12 months, were chosen based on yield-related traits, and the age difference between the breeds is not significant. All animals were reared locally by farmers under similar extensive farming management systems typical of the region, with communal free-range grazing. Routine health management practices, such as deworming and vaccination, were followed according to the recommendations of local veterinary doctors. All animals underwent a 24-hour fasting period with ad libitum access to water to reduce stress and ensure standardisation in pre-slaughter metabolic conditions. After weighing, animals were then scientifically slaughtered at ICAR-NMRI (FSSAI-approved slaughterhouse) by stunning and following animal welfare guidelines. The tail, head, skin, hooves, and viscera were removed after exsanguinating.

### Measurement of meat quality indices

The *longissimus dorsi* muscle was collected after chilling the carcass for 24 hours to measure the meat quality indices.

#### Water holding capacity

To determine the water holding capacity (WHC), approximately 10 grams of the minced muscle was mixed with 15 mL of 0.6 M NaCl solution in a 50 mL centrifuge tube using the tissue homogeniser [21]. The mixture was then incubated for 15 minutes at 4^o^C. Afterwards, the sample was centrifuged at 1,081×g at 4°C for 15 minutes using a refrigerated centrifuge (Himac; Eppendorf). The WHC was calculated by measuring the volume of solution retained by meat, and reported as a percentage of the total volume, using the formula below.


(1) $$\text{WHC}=\frac{\text{Volume of Nacl taken}-\text{Measured volume of NaCl after centrifugation}}{\text{Weight of the Sample}}\times 100$$

#### pH measurement

The pH of the LD muscle (24 h) was analysed by homogenising 5 grams of meat with 45 mL of distilled water using a tissue homogeniser (T 18 digital ULTRA-TURRAX; IKA). The pH was determined using a calibrated pH electrode meter (FP20 BIOKIT; Mettler Toledo).

#### Colour measurement

The colour of the LD muscle (24 h) was measured by employing a calibrated colourimeter (CR-20; Konica Minolta). The calibration was performed using a standard white tile. Afterwards, the surface aperture of the colourimeter was positioned on the surface of the muscle at five different locations to obtain the readings for lightness (L*), redness (a*) and yellowness (b*) as per Commission Internationale de l’Eclairage (CIE).

#### Warner-Bratzler shear force and cooking loss

The *longissimus dorsi* muscle samples were collected after 24h of carcass chilling, sealed in polythene pouches, and cooked in a water bath at a temperature of 100^o^C until the internal temperature reached 75°C. The samples were weighed before and after cooking, and the cooking loss was calculated as the % of weight loss between the two measurements. The post-cooking samples were refrigerated at 4°C±1°C for 24 hours and then allowed to return to room temperature. From each sample, three cylindrical cores of 1.25 cm diameter were taken in parallel to the orientation of the muscle fibres using a tissue borer. These cores were subsequently sheared perpendicular to the muscle fibres using a texture analyser (H1KF; Tinius Olsen) equipped with a Warner-Bratzler blade (V-shaped, 60° angle). The machine was operated at a crosshead speed of 200 mm/min and a load force of 75 N.

#### Masson trichome staining

Masson trichome staining was performed as described by Zhao et al [22] with some modifications. Briefly, *longissimus dorsi* muscle samples fixed in neutral buffer formalin (NBF) were paraffin-embedded, sectioned at 5 μm and stained to visualise muscle fibres (red), nuclei (black), and collagen (blue) under a light microscope. Ten random fields per sample were analysed for collagen content using ImageJ software, and the collagen content was expressed as the percentage of the area occupied by blue-stained pixels.

#### Texture profile analysis

The texture of the meat samples was analysed according to the method described by Huang et al [23], with some modifications. Samples were prepared to standard dimensions of 4 cm (length)×4 cm (width)×3 cm (thickness) to ensure consistency across measurements. A Rapid TA Max Texture analyser (Shanghai Tengba Instrument Technology) fitted with a compression plate attachment (PL100 – Plate probe) with a test speed of 3mm/s, probe speed of 6mm/s, measuring time of 3 s, a trigger force of 5 N, and each sample was subjected to two cycles of 50% compression of their original height. The parameters determined included hardness, springiness, cohesiveness, gumminess, chewiness and resilience.

### Nutritional quality determination

#### Proximate composition analysis

The differences in compositional parameters, such as fat, moisture, ash, and protein content, were analysed according to the AOAC protocols. Specifically, the moisture content was determined using the hot air oven method (AOAC 950.46), crude protein by the Kjeldahl method (AOAC 990.03), and fat by the Soxhlet extraction method (AOAC 991.36).

#### Fatty acid composition analysis

The fatty acid composition was analysed according to P [24], and the fatty acid methyl esters (FAMEs) were prepared from the fat extracted from the intramuscular region. One gram of the sample was mixed with 2 mL of a 14% (w/w) boron trifluoride-methanol reagent, and the mixture was heated at 60°C in a water bath for 1 hour. After cooling, 2 mL of hexane and 5 mL of deionised distilled water were added, and the mixture was centrifuged at 1,021×g for 10 min. The upper hexane layer containing FAMEs was transferred to a vial for GC-FID analysis. FAMEs were separated by gas chromatograph (7820A; Agilent Technologies) using SP 2560 capillary column (100 m×0.25 mm×0.2 μm). The oven temperature was programmed to start at 100°C for 5 minutes, increase to 240°C at 4°C/min, and hold for 20 minutes. The injector temperature was set at 210^o^C, and the nitrogen was used as the carrier gas at 1mL/min. FAMES were identified by comparing the retention times with a 37-component FAME standard (Supelco). Finally, the composition of the fatty acids was calculated based on peak area and expressed as a percentage of total fatty acids. The individual fatty acid content was estimated using the conversion factor of 0.953 [24].

### RNA extraction and cDNA synthesis

The total RNA was extracted from meat samples using the GENEZol reagent according to the manufacturer’s guidelines. Briefly, 50 mg of *longissimus dorsi* muscle was homogenised in 1 mL of GENEZol reagent and incubated for 5 min at room temperature. The mixture was centrifuged at 15,000×g for 10 min, and the clear Supernatant was carefully collected. To this, 200 μL of chloroform was added per 1 mL GENEZol reagent was added, followed by vigorous shaking for phase separation. Then centrifuged at 15,000×g for 15 minutes at 4°C. The upper aqueous phase, containing the RNA, was transferred to a new tube, mixed with an equal volume of isopropanol, and incubated for 10 min at room temperature. Afterwards, centrifuged at 14,000×g for 15 min at 4°C to form a tight RNA pellet; the pellet was washed with 1ml of 70% ethanol and centrifuged again as mentioned above. The RNA pellet was air-dried and resuspended in 30 μL of RNase-free water, followed by incubation at 58°C for 10 min. The RNA was stored at −40°C until cDNA synthesis.

The RNA concentration and quality were measured using the IMPLEN nanophotometer. Reverse transcription of mRNAs was carried out with the iScript cDNA synthesis Kit (Bio-Rad) as per the manufacturer’s protocol. The reverse transcription polymerase chain reaction (RT-PCR) was conducted in a 20 μL reaction volume, with 200 ng of RNA template. The RT reaction mix consisted of 4 μL of 5x reaction buffer, 1 μL enzyme mix, and 13 μL RNase-free water (Bio-Rad). This reaction was carried out in the Thermal cycler (C 1000 Touch; Bio-Rad) using the following conditions: Priming at 25°C for 5 min, Reverse transcription at 46°C for 20 min and RT inactivation at 95°C for 1 min. Then, the cDNA was stored at −40°C until the quantitative real-time PCR assay was performed.

### Quantitative real-time polymerase chain reaction

Expression levels of candidate genes were analysed using SYBR Green dye-based quantitative real-time PCR (qRT-PCR) assay. The ovine candidate gene-specific primers were selected from the literature (Table 1). The assay was standardised by optimising the PCR cycle number (35, 40, 45, 50), annealing temperature (55°C to 72°C) and concentration of primers (200 nM to 600 nM) in accordance with MIQE (Minimum Information for Publication of Quantitative Real-Time PCR Experiments) guidelines. The final reactions were performed in a total volume of 10 μL, using iTaq Universal SYBR Green Supermix (Bio-Rad). Hard-Shell 96-Well PCR Plates (Bio-Rad) were used to prepare and properly mix the reaction mixture. Each reaction was performed in triplicates along with all the controls (positive & negative). The average of these replicates was used to evaluate the relative amount of the targeted candidate gene. GAPDH was used as a reference gene for the normalisation of the results based on its reported stability in ovine skeletal muscle tissue. A translucent Microseal adhesive sealing film was used to seal the plate. The contents of each well were mixed at 500 rpm using a Thermomixer (Eppendorf, USA) and then centrifuged for 5 minutes at 629×g to remove the air bubbles. The PCR was conducted under the following conditions

To ensure the accuracy of the PCR results, a melt curve was inserted over a temperature range from 65°C to 95°C and a ramp rate of 1.6°C/s. the specificity of the amplification products and the absence of primer dimers were verified using melt curve analysis. Following the real-time PCR run, Cq values were quantified by performing the baseline adjustments according to the standard rule (i.e., one-third of the endpoint RFU) to ensure the common threshold across all samples. After that, the relative fold change (2-ΔΔCq) in gene expression for all the groups was calculated relative to the group with the highest Cq value [25].

### Statistical analysis

Data analysis was conducted using Graphpad Prism. Data normality was assessed before performing the Student t-test to confirm the appropriateness of parametric testing. A student unpaired two-tailed (parametric) t-test (n = 4) was conducted to compare the indices among the different breeds under investigation. Pearson’s correlation analysis and matrices are drawn using SRPLOT. The findings were presented as mean values along with their corresponding standard errors. Statistical differences between groups were considered significant when the p-value was less than or equal to 0.05.

## RESULTS

### Proximate composition

The differences in compositional parameters of the *longissimus dorsi* muscle are summarised in Table 2. Moisture content was slightly but significantly higher in Deccani breed (75.57±0.35) compared with the Nellore breed (74.05±0.42, p = 0.0321). Similarly, the fat (IMF) was higher in the Deccani breed (4.39±0.23) than in Nellore breed (3.29±0.21, p = 0.0119). In contrast crude protein was marginally higher in Nellore breed (21.98±0.60) compared with Deccani breed (19.56±0.26, p = 0.0105). However, there is no significant difference was observed in the ash content between the breeds.

### Fatty acid composition

Significant breed-related differences in the fatty acid composition of *longissimus dorsi* muscle were observed for several fatty acid groups in Table 3. The Deccani breed exhibited significantly higher proportions of SFA, unsaturated fatty acids (UFA), MUFA, and polyunsaturated fatty acids (PUFA) compared to the Nellore breed. Likewise, the proportion of desirable fatty acids and the desaturase activity index were significantly greater in the Deccani breed, indicating a more active enzymatic conversion of saturated to UFA in this breed. In contrast, the atherogenic index, which reflects the potential dietary risk factors associated with cardiovascular health, did not differ significantly between breeds (p>0.05). Similarly, lipid quality ratios showed no statistically significant differences, although numerical variations were noted. The Deccani breed tended to exhibit higher UFA/SFA and PUFA/SFA ratios, whereas the Nellore breed showed a slightly higher MUFA/PUFA ratio.

### Texture profile analysis

The instrumental texture profile revealed subtle breed-related differences as depicted in Table 4. Meat from the Deccani breed exhibited significantly lower chewiness (537.53±62.30) and gumminess (864.76±80.37) compared to the Nellore breed (786.04±33.06 and 1,204.40 ±69.49, respectively), with a p-value for chewiness being 0.0244 and for gumminess being 0.0330. These results suggest that Deccani meat had a softer and less resistant texture in mastication. Cohesiveness and springiness did not vary significantly across the breeds (p>0.05).

### Breed-wise comparison of meat quality traits

**Figure 1:**
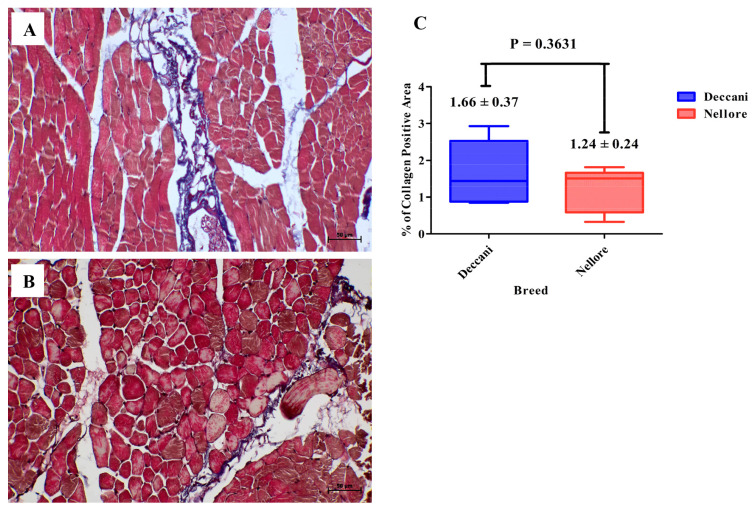
Collagen content in *longissimus dorsi* muscle tissue of Deccani and Nellore sheep breeds assessed by Masson’s trichome staining. (A) Representative histological section from Deccani muscle tissue showing extensive blue-stained collagen deposition between muscle fibres, indicating higher collagen content. (B) Representative histological section from Nellore muscle tissue showing visibly reduced blue-stained collagen, suggesting lower collagen accumulation (Masson's Trichrome staining). (C) Quantitative comparison of collagen-positive area (%) between breeds. Box plot displays mean±standard error for Deccani and Nellore breeds.

**Figure 2:**
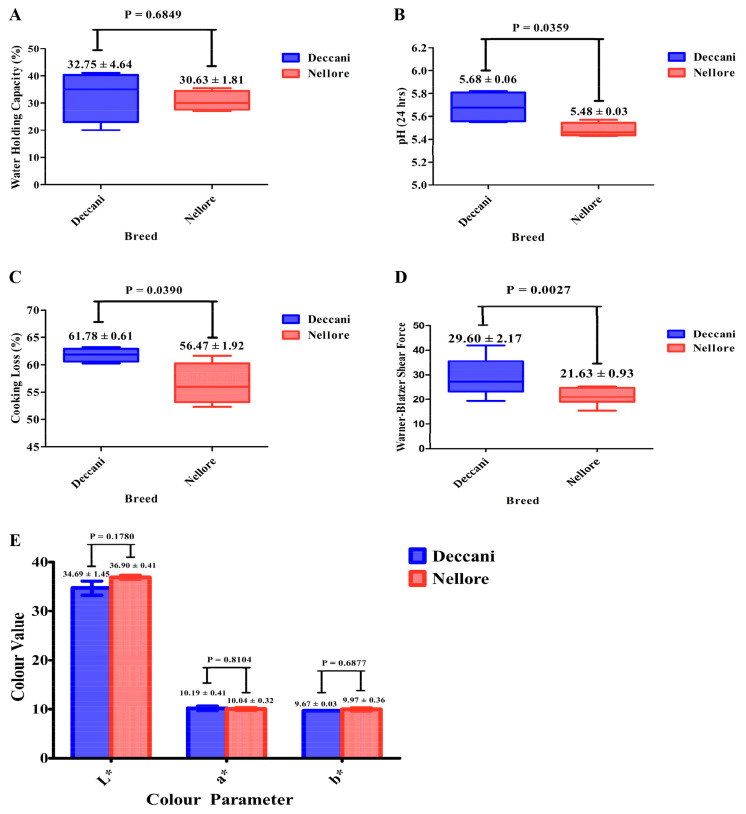
Comparative graphical representation of (mean±standard error and p-values) of meat quality attributes of *longissimus dorsi* muscle between Deccani and Nellore sheep breeds. (A) Box plots illustrating WHC, showing breed-wise variation in moisture retention. (B) muscle pH measured at 24 hours of postmortem, reflecting differences in postmortem biochemical changes across the breeds. (C) Warner-Bratzler shear force values, indicating variation in meat tenderness between breeds. (D) Cooking loss percentages representing breed-specific differences in moisture retention during thermal processing. (E) Bar graph comparison of meat colour parameters, including lightness (L*), redness (a*), and Yellowness (b*), between Deccani and Nellore breeds. WHC, water holding capacity.

Breed-specific differences were observed across multiple meat quality parameters, such as WHC, pH (24 h), WB shear force, cooking loss, colour attributes, and collagen content. No significant differences were observed in Collagen content (1.66±0.37 vs. 1.24±0.24, 0.3631) on Masson’s trichome staining under a light microscope, as shown in Figure 1. WHC tended to be higher in the Deccani breed (32.75±4.64) compared with the Nellore breed (30.63±1.81), but the difference was not statistically significant (p = 0.6849), as shown in Figure 2A. However, significant differences were observed in ultimate pH, cooking loss and tenderness. Deccani meat had a slightly higher ultimate pH at 24 h postmortem (5.68±0.06) compared with the Nellore breed (5.48±0.03, p = 0.0359), as shown in Figure 2B. Cooking loss percentage was significantly lower in the Nellore breed (54.47±1.92, p = 0.0390) than the Deccani breed (61.78±0.61), suggesting improved juiciness and reduced exudate during cooking (Figure 2C). Tenderness, assessed by WBSF, further supported this pattern represented in Figure 2D. Nellore breed exhibited significantly lower shear force values (21.63±0.93, p = 0.0027) than Deccani (29.60± 2.17). Further, meat colour (L*, a*, b* values) did not differ significantly between the breeds (Figure 2E).

**Figure 3:**
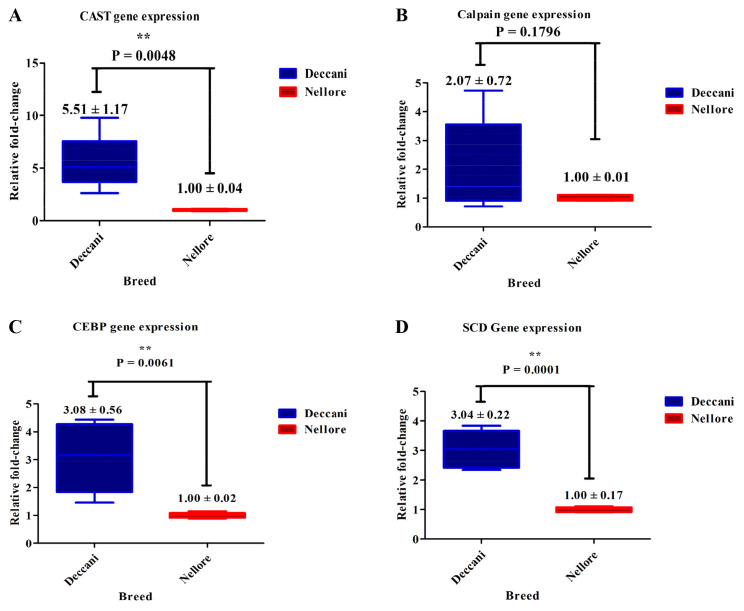
Relative gene expression levels (mean±standard error and p-values) of tenderness, IMF, and FA composition-related genes in the *longissimus dorsi* muscle tissue of Deccani and Nellore sheep breeds. (A) Comparison of CAST gene expression in Deccani relative to Nellore. (B) Comparison of CAPN1 gene expression levels illustrating breed-wise variation. (C) Comparison of CEBPB gene expression levels in Deccani relative to Nellore. (D) SCD gene expression in Deccani relative to Nellore. IMF, intramuscular fat.

Expression analysis of four candidate genes (CAST, CAPN, SCD, and CEBPB) in terms of mRNA is shown in Figure 3. Relative expression of the CAST gene was 5.51-fold higher in the Deccani breed compared to the Nellore breed (p<0.05). The Deccani breed also showed a significantly higher level of CEBPB mRNA than the Nellore (p<0.05), and SCD mRNA abundance was significantly different between the Deccani and Nellore breeds (p<0.05). Moreover, no statistical significance was observed in the expression of CAPN between the two breeds (p>0.05).

### Correlation analysis of gene expression and meat quality traits

**Figure 4:**
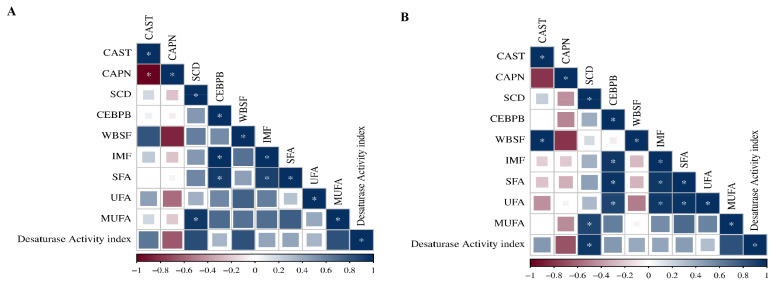
Correlation matrices showing relationships among gene expression markers, meat quality traits, and fatty acid composition in Nellore and Deccani sheep breeds. (A) represents Matrix correlations in Nellore samples, while (B) shows Matrix correlations in Deccani samples. Each cell displays the pairwise Pearson correlation coefficient between variables, including CAST, CAPN, SCD, CEBPB, Warner-Bratzler shear force (WBSF), intramuscular fat (IMF), saturated fatty acids (SFA), unsaturated fatty acids (UFA), monounsaturated fatty acids (MUFA) and Desaturase Activity index. Red colour represents negative correlation, blue colour represents positive correlation, and white colour represents no correlation. Asterisks (*) indicate statistically significant correlations.

Distinct patterns of association were observed between the two breeds, indicating breed-specific interactions among genetic factors and meat quality attributes. Pearson correlation analysis between gene expression and phenotypic traits for each breed is presented in Figure 4. In the Deccani breed, IMF content showed a strong positive correlation (r = 0.95, p = 0.02) with CEBPB expression and a weak positive correlation (r = 0.37, p = 0.54) with SCD. IMF also showed a weak negative correlation with CAST (r = −0.18, p>0.05), CAPN (r = −2.1, p>0.05), and WBSF (r = −0.77, p>0.05). WBSF positively correlated with CAST (r = 0.76, p>0.05) and negatively correlated with CAPN (r = −0.79, p>0.05). Fatty acid composition, MUFA (r = 0.90, p = 0.04), and desaturase activity index (r = 0.95, p = 0.01) showed a strong positive correlation with the SCD expression, while SFA showed significant positive correlations with the CEBPB expression (r = 0.97, p = 0.01 and IMF content (r = 0.95, p = 0.01). Furthermore, in the Nellore breed, WBSF exhibited a strong negative correlation (r = −0.98, p = 0.003) with CAPN expression, and a positive correlation (r = 0.82, p>0.05) with CAST. CEBPB expression showed a significant positive correlation with IMF (r = 0.98, p = 0.003) and SFA (r = 0.91, p = 0.03) content, whereas SCD displayed a strong positive correlation (r = 0.96, p = 0.01) with MUFA, and a non-significant positive correlation (r = 0.85, p = 0.07) with desaturase activity index.

## DISCUSSION

The present study provides a comprehensive evaluation of breed-related differences in carcass characteristics, proximate composition, meat quality traits, fatty acid profiles, texture attributes, and the expression of key candidate genes in sheep. By combining phenotypic measurements with molecular data, the findings highlight the complex interplay of genetics, muscle biochemistry, and carcass traits in determining meat quality outcomes.

Higher IMF content in the Deccani breed was positively correlated with elevated expression of CEBPB, a transcription factor central to adipocyte differentiation and lipid biosynthesis. This finding is biologically consistent with the role of CEBPB in promoting marbling, which in turn enhances juiciness and flavour, as highlighted by Pannier et al [26]. Nellore breed, by contrast, had relatively higher protein content, reflecting a leaner carcass phenotype that was not accompanied by strong upregulation of adipogenic genes. Ash content did not differ between breeds, confirming earlier observations that mineral content is relatively stable across genotypes [27]. Despite its higher IMF, the Deccani exhibited greater WBSF, indicating tougher meat. The weak negative correlation of IMF with CAST, CAPN and WBSF suggests that greater IMF was generally associated with lower shear force. Typically, IMF is associated with improved tenderness, yet this expected relationship was overridden here by molecular regulation. The Deccani breed expressed significantly higher levels of CAST, the endogenous inhibitor of CAPN proteases, which suppresses postmortem myofibrillar proteolysis. The strong positive correlation between CAST expression and WBSF in our data supports the interpretation that elevated CAST activity delayed tenderization, counteracting the potential softening of higher IMF. In contrast, CAPN expression did not differ significantly between breeds, suggesting that the limiting factor for tenderness variation was CAST, rather than calpain activity. Similar findings were noted by Hopkins et al [28], who emphasised the calpain-calpastatin balance as a determinant of tenderness variability. Texture profile analysis further reinforced these contradictions. Deccani meat exhibited lower chewiness and gumminess, despite being tougher by WBSF. This suggests that while CAST-driven inhibition of proteolysis limited tenderization, structural attributes such as connective tissue cohesiveness and fat distribution reduced mastication resistance. Such multidimensional differences highlight that tenderness cannot be fully explained by IMF or shear force alone, a point emphasised in consumer studies where different texture parameters contribute variably to eating quality [26].

Deccani contained significantly higher levels of total SFA, UFA, MUFA, PUFA, as well as desirable fatty acids and desaturase activity index, reflecting a strong lipogenic phenotype. These results correspond with the upregulation of CEBP and SCD, which encodes stearoyl-CoA desaturase, a key enzyme in the conversion of SFA to MUFA. The positive correlations between SCD expression and MUFA in our study provide mechanistic support for the role of SCD in enhancing lipid quality. Similar associations were reported by Fan et al [29], who identified SCD as a major regulator of fatty acid composition in sheep *longissimus dorsi* muscle. The Nellore breed exhibited lower expression of lipogenic genes and correspondingly lower fat deposition. Despite this, health-related indices such as PUFA/SFA ratio, MUFA/PUFA ratio, and atherogenic index did not differ significantly between breeds. This suggests that, despite having more fat overall, the relative proportions of healthy vs. unhealthy fatty acids were balanced. Wang et al [30] similarly reported that while feeding regimens could shift the absolute levels of SFA, MUFA, and PUFA in sheep, the derived nutritional ratios often remained relatively stable, reflecting the resilience of these indices across both management systems and genetic backgrounds.

The contradictions that arose in this study underscore the critical role of gene expression in determining whether favourable carcass and compositional traits translate into superior eating quality. Importantly, the discrepancies revealed here are not biological dead-ends but opportunities. If the expression of the CAST gene can be reduced through nutritional interventions, feeding systems, or selective breeding and if CAPN activity can be enhanced, the tenderness limitation of high IMF breeds could be overcome. Similarly, further optimisation of SCD and CEBPB expression may enhance IMF and fatty acid compositional quality in leaner breeds without compromising the tenderness. By framing gene expression as a modifiable pathway rather than a fixed outcome, this study highlights the potential for targeted strategies to reconcile productivity, eating quality and nutritional value in Indian sheep production. Recent studies have reported that nutritional interventions altered the meat quality traits and growth performance in sheep [31,32]. Such approaches, integrating breeding, feeding and molecular tools, will be crucial for delivering consistent, high-quality sheep meat that meets both industry demands and consumer expectations. However, in the present study, a limited number of animals were used. This may limit the broader generalisation of the findings. Despite this, well-defined biological and technical replicates, along with an integrated analysis of meat quality traits and gene expression, provided meaningful insights into breed-associated differences. Further studies with larger sample sizes are needed.

## CONCLUSION

This study demonstrated clear differences between the two sheep breeds evaluated under extensive feeding systems. The Deccani breed offers superior IMF and flavour potential, along with a high amount of desirable fatty acids and lower chewiness and gumminess, resulting in a more nutritious quality and enjoyable mouthfeel during consumption. Conversely, the Nellore breed’s balanced proteolytic activity and leaner composition made it favourable for tenderness and yield, which has direct economic significance for both the producers and consumers. Strategic modulation of CAST, CAPN, SCD, and CEBPB expression could help in developing superior genotypes suited for both consumer preference and industry needs.

## Figures and Tables

**Table 1:** List of primer sequences used for relative quantification by real-time PCR

Gene name	Primer sequence	Amplicon size	Reference
*CAST*	F- 5′-TGCCCTGGATCAACTTTCTG-3′	149	Bagatoli et al [14]
	R- 5′-GGTATTTAGGTGGGATGGTGTC-3′		
*CAPN1*	F- 5′-AACATTCTCAACAAAGTGGTG-3′	190	Dervishi et al [18]
	R- 5′-ACATCCATACAGCCACCAT-3′		
*SCD*	F - 5′-CCCAGCTGTCAGAGAAAAGG-3′	115	Dervishi et al [18]
	R - 5′-GATGAAGCACAACAGCAGGA-3′		
*CEBPB*	F - 5′-GACAAGCACAGCGACGAGT-3′	102	Dervishi et al [18]
	R - 5′-GTGCTGCGTCTCCAGGTT-3′		
*GAPDH*	F - 5′-CCAGGCAGAGAACGGGAAG-3′	78	Bagatoli et al [14]
	R - 5′-GCCTTCTCCATGGTAGTGAAG-3′		

PCR, polymerase chain reaction.

**Table 2:** Differences in major compositional parameters in Deccani and Nellore sheep (measured in the *longissimus dorsi* muscle)

Sl. No.	Parameter	Deccani	Nellore	p-value
1.	Protein	19.56±0.26	21.98±0.60	0.0105
2.	Fat	4.39±0.23	3.29±0.21	0.0119
3.	Moisture	75.57±0.35	74.05±0.42	0.0321
4.	Ash	0.80±0.04	0.83±0.02	0.6919

**Table 3:** Differences in major fatty acid compositions in Deccani and Nellore sheep breeds (measured in the *longissimus dorsi* muscle)

Sl. No.	Type/Name of the fatty acid	Nellore (n = 4)	Deccani (n = 4)	p-value
1.	Caprylic acid (C8:0)	1.19±0.02	<0.1 (BLOQ)	
2.	Caproic acid (C10:0)	0.11±0.01	0.16±0.01	0.0846
3.	Lauric acid (C12:0)	0.30±0.12	0.44±0.18	0.5742
4.	Myristic acid (C14:0)	1.40±0.10	3.47±0.33	0.0272
5.	Palmitic acid (C16:0)	20.06±0.44	24.63±0.67	0.0294
6.	Margaric acid (C17:0)	1.07±0.29	1.56±0.17	0.2819
7.	Stearic acid (C18:0)	18.11±0.81	23.33±0.49	0.0314
8.	Lignoceric acid (C24:0)	0.44±0.09	<0.1 (BLOQ)	
9.	Palmitoleic acid (C16:1)	1.11±0.10	1.24±0.42	0.7985
10.	10 C Heptadecanoic acid (C17:1)	0.71±0.02	0.48±0.04	0.0367
11.	Oleic acid (C18:1)	19.44±0.40	25.15±1.11	0.0400
12.	Nervonic acid (C24:1)	1.21±0.05	<0.1(BLOQ)	
13.	Linolenic acid (C18:2)	1.50±0.07	0.88±0.03	0.0181
14.	Y-Linolenic acid (C18:3) PUFA	0.11±0.00	0.23±0.01	0.0191
15.	Cis-13,16-docosadienoic acid (C22:2)	6.98±0.43	11.64±0.61	0.0249
16.	Saturated fatty acids	42.66±0.58	53.59±0.51	0.0050
17.	Unsaturated fatty acids	30.56±0.14	40.12±1.26	0.0171
18.	Monounsaturated fatty acids (MUFA)	22.46±0.54	26.87±0.73	0.0398
19.	Polyunsaturated fatty acids (PUFA)	8.09±0.38	13.26±0.52	0.0155
20.	UFA/SFA	0.64±0.06	0.75±0.03	0.2427
21.	MUFA/PUFA	2.78±0.20	2.02±0.02	0.0645
22.	PUFA/SFA	0.19±0.01	0.25±0.01	0.0513
23.	Desirable fatty acids	48.67±0.66	63.45±1.75	0.0157
24.	Atherogenic index (AI)	0.93±0.02	1.09±0.12	0.2313
25.	Desaturase activity index	0.45±0.01	0.59±0.01	0.0174

BLOQ – Below the limit of quantification; Atherogenic index (AI: [12:0+4 X 14:0+16:0]/Sn–6+Sn–3+SMUFA) [33].

D-9 desaturase activity index for 18:0 was estimated using the following ratio: 18:1/(18:0+18:1) [34].

Desirable fatty acids (MUFA+PUFA+18:0) [35].

**Table 4:** Differences in parameters of texture profile analysis in Deccani and Nellore Sheep breeds (measured in the *longissimus dorsi* muscle)

Sl. No.	Parameter	Deccani (n = 4)	Nellore (n = 4)	p-value
1.	Hardness	1,280±156.1	1,769±116.0	0.0655
2.	Springiness	0.63±0.06	0.65±0.01	0.6820
3.	Chewiness	537.53±62.30	786.04±33.06	0.0244
4.	Gumminess	864.76±80.37	1,204.40±69.49	0.0330
5.	Cohesiveness	0.68±0.05	0.68±0.01	0.9488

## Data Availability

Upon reasonable request, the datasets of this study can be available from the corresponding author.
